# Assessing *Aedes* mosquito larval indicators, dengue virus infection rates, and risk factors in Khyber Pakhtunkhwa: Insights for improved vector control strategies

**DOI:** 10.1371/journal.pntd.0013252

**Published:** 2025-07-22

**Authors:** Jehangir Khan, Muhammad Adil, Tsheten Tsheten, Pablo Manrique-Saide, Dongjing Zhang, Abdul Aziz, Zhiyue Lv, Tao Chen

**Affiliations:** 1 Hainan General Hospital, Hainan Affiliated Hospital of Hainan Medical University, Haikou, Hainan, China; 2 Key Laboratory of Tropical Disease Control (Sun Yat-sen University), Ministry of Education, Guangzhou, Guangdong, China; 3 Zoology Department, Abdul Wali Khan University Mardan, Khyber Pakhtunkhwa, Pakistan; 4 Pakistan Bureau of Statistics, Islamabad, Pakistan; 5 National Centre for Epidemiology and Population Health, Australian National University, Australia; 6 Unidad Colaborativa para Bioensayos Entomologicos y Laboratorio para el Control Biologico de Aedes aegypti, Campus de Ciencias Biologicas y Agropecuarias, Universidad Autonoma de Yucatan, Mérida, Mexico; 7 Chinese Atomic Energy Agency Center of Excellence on Nuclear Technology Applications for Insect Control, Key Laboratory of Tropical Disease Control of the Ministry of Education, Sun Yat-sen University, Guangzhou, China; 8 International Atomic Energy Agency Collaborating Centre, Sun Yat-sen University, Guangzhou, China; 9 Nuclear Institute for Food and Agriculture (NIFA), Peshawar, Khyber Pakhtunkhwa, Pakistan; 10 Hainan Provincial Bureau of Disease Prevention and Control, Haikou, China; Egerton University, KENYA

## Abstract

**Background:**

Effective dengue management hinges on targeting key vector breeding sites and understanding transmission risks. Despite recurring outbreaks in Pakistan’s Khyber Pakhtunkhwa (KP) Province since 2013, comprehensive entomological and virological data remain scarce. This study identified key larval-based indicators (habitats, *Stegomyia* indices), mosquito species composition, and dengue virus (DENV) infection rates in *Aedes* mosquitoes, evaluating their contributions to outbreak risk.

**Methodology/principal findings:**

From July to December 2021, a cross-sectional larval survey of *Aedes aegypti* and *Ae. albopictus* was conducted across epidemiologically high-risk KP districts, inspecting water-holding containers located indoors, outdoors, and on rooftops. Additionally, adult mosquitoes were collected using aspirators and nets, with weekly dengue case data sourced from Peshawar’s Directorate of Health Services. A subsample of 200 adult mosquito pools (20 per district) underwent RT-PCR to determine minimum infection rates (MIR). Larval indices revealed a House Index (HI) of 19.4%, a Container Index (CI) of 20.4%, and a Breteau Index (BI) of 89%. *Aedes aegypti* was the dominant species, accounting for 62% of larvae and 67.8% of adult mosquitoes. Peshawar (BI = 89.3), Nowshera (BI = 71.4), and Mardan (BI = 57) reported the highest Breteau indices and corresponding dengue case counts: 2,584 (48.8%), 404 (7.6%), and 327 (6.2%), respectively. The peak larval positivity was recorded in October (29.3%) and September (24.7%), aligning with dengue patient hospitalization rates of 52.8% and 46.8%, respectively. Common breeding sites included indoor flowerpots (25.4%), outdoor rubber tyres (16%), and roof tap water (23.7%). Container type and location significantly (P < 0.00) predicted larval abundance. Regression analysis revealed significant associations between dengue incidence, population density, and *stegomyia* indices. Of 38 positive pools (19%), DENV-2 and DENV-3 predominated (47.4% each), with peak MIRs recorded in Peshawar (30), Mardan (25), and Haripur (25).

**Conclusions/significance:**

High larval indices and dual-serotype circulation in adaptable *Aedes* vectors signal substantial outbreak risk in KP. These findings underscore the need for targeted vector strategies, focusing on containers with the highest breeding potentials and epidemiological significance, particularly in high-transmission areas. Further molecular and entomological investigations are critical to corroborate these findings and inform more effective interventions.

## Author summary

Since 2013, dengue has spread rapidly throughout KP Province, northern Pakistan, resulting in major outbreaks with over 20,000 reported cases and nearly 100 fatalities. *Ae. aegypti* is the primary dengue vector, with *Ae. albopictus* emerging as a new concern. This study addresses the significant gaps in entomological and molecular surveillance by identifying key dengue risk factors across multiple districts in KP. We conducted surveys of water-holding containers in households to detect *Ae. aegypti* and *Ae. albopictus* larvae, assessed mosquito infection rates with DENV, and calculated *Stegomyia* indices to measure infestation levels. Our findings show that *Ae. aegypti* was the dominant vector, making up about 67% of all samples, while *Ae. albopictus* displayed considerable adaptability to both domestic and peri-domestic environments. Metropolitan districts such as Peshawar, Mardan, and Nowshera exhibited alarmingly high levels of *Aedes* infestation, with Breteau Indices of 89.3, 71.4, and 57, respectively. Molecular analysis revealed that 19% of mosquito pools were infected with DENV-2 and DENV-3, underscoring the ongoing transmission of multiple dengue virus serotypes. These findings highlight the urgent need for targeted vector control strategies and continuous entomological surveillance to address the growing public health threat posed by dengue in the region. By providing critical baseline data, this study supports the development of cost-effective, region-specific interventions to reduce dengue transmission and prevent future outbreaks.

## Introduction

Dengue fever affects over 129 countries, with cases escalating from 0.51 million in 2000 to 4.2 million in 2019, over 70% of which occur in Asia [1]. In Pakistan, dengue is believed to have been introduced to Karachi in 1994 via tires with DENV-infected *Aedes* mosquito eggs from India, where the first epidemic, with about 57 cases, was reported that year [2,3]. Since 1994, Pakistan has experienced nationwide dengue outbreaks, resulting in 300,262 reported cases and 1,115 deaths by 2022 [1].

*Ae. aegypti* and *Ae. albopictus* are the two main DENV mosquito vectors worldwide. Both species colonise and develop in water-holding containers in a variety of environments (rural, urban, and semi-urban, public and residential), laying eggs in both natural and manmade habitats [3,4]. *Ae. aegypti* feeds on humans and prefers to stay indoors, whereas *Ae. albopictus* feeds on humans and other animals and likes to stay outside. *Ae. aegypti* was first reported in Pakistan between 1969 and 1971, and *Ae. albopictus* since 2000 [3]. Extensive movements of internally displaced people (IDPs) due to military operations, flooding, and earthquakes, combined with increased urbanization over the past 14 years, has led to slum settlements with inadequate water, sanitation, and waste management facilities, creating numerous new breeding habitats for both vector species [1,5]. The climate of some regions in Pakistan also fosters mosquito breeding sites because of favourable tropical rainfall, humidity, and temperature [5]. As a result, mosquito vectors have increasingly invaded new locations, significantly raising the risk of dengue transmission.

Because of the limitations of vaccine and treatment options for dengue, vector management remains the primary strategy to limit disease spread. In dengue-endemic areas, WHO recommends routine vector surveillance to assess changes in vector populations and their distribution, aiming to anticipate outbreaks and monitor control efforts [6–8]. *Stegomyia* indices (house index [HI], container index [CI], and Breteau index [BI]) are recommended for entomological surveillance, as they help set thresholds to prevent dengue transmission and link vector density to disease risk [9,10]. The use of larval (*Aedes*) indices with well-established threshold values at the national level may improve efficient entomological dengue control by providing accurate early warnings of impending epidemics [7,11–13]. There are more opportunities for clustered transmission of dengue virus when higher densities of *Aedes* mosquitoes exist in or around a household [14,15]. Thus, by exploring *Aedes* infestation ‘hot spots’, targeted vector management could be an effective strategy in the case of limited intervention resources [16]. Moreover, dengue virus infection modifies mosquito behaviour, making them more attracted to hosts for blood feeding, thereby increasing their vectoral capacity [17]. Dengue epidemiology could be significantly impacted by DENV-induced alteration in mosquitoes’ host-seeking and biting behaviours [17]. Thus, monitoring of mosquito-virus infection rates can offer significant predictive indicators of virus transmission patterns corresponding to higher human risk [18]. However, very limited entomological and molecular studies have been conducted so far in Pakistan.

Dengue is spreading to new locations in Pakistan, exacerbating the public health problem and healthcare service delivery. There is limited knowledge examining the entomological parameters that contribute to the transmission of dengue fever. This study will serve as a baseline in the country to understand and recognize: (i) vector mosquito larval infestation levels (vector indices), preferred breeding sites (container types), and species composition across the province; (ii) molecular characterization of DENV in adult *Aedes* vector; and (iii) associations between the larval indices, MIRs, and the dengue transmission risk. Given the ongoing challenges of dengue control in KP Province, this study aims to generate data that can inform the development of targeted, cost-effective vector control programs, enhancing public health outcomes.

## Methods and materials

### Ethical statement

The study followed ethical standards in accordance with the general guidelines of the Zoology Faculty, AWKUM, and national field sampling protocols, with prior informed consent obtained from household residents [1,19].

### Study area

**Fig 1:**
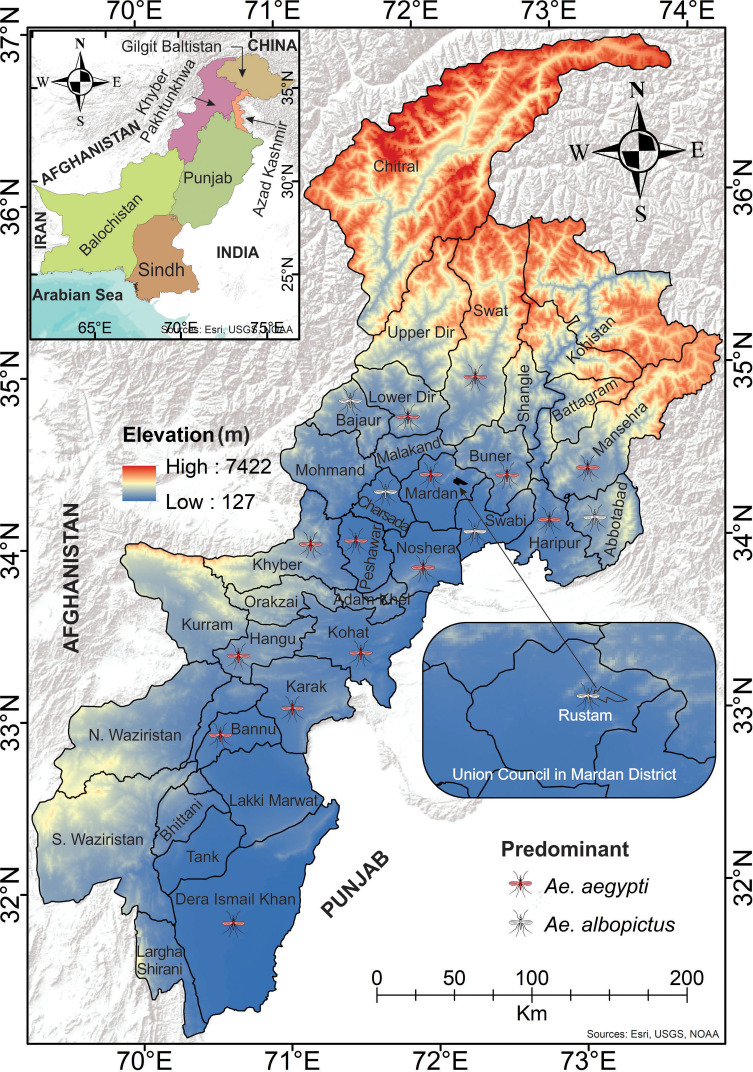
Map of Khyber Pakhtunkhwa (KP), Pakistan, showing the sampled districts for *Aedes* mosquito collection. The elevation gradient (meters) is represented, with lower elevations in blue and higher elevations in red. The mosquito icons indicate the districts where sampling was conducted. The inset map in the upper left highlights KP within Pakistan, while the bottom right inset zooms in on Rustam, a Union Council in the Mardan District. Data sources: DIVA-GIS (https://diva-gis.org/).

Khyber Pakhtunkhwa (KP: 34.9526°N, 72.3311°E) (Fig 1) is a geographically and climatically diverse province in northwestern Pakistan [20]. Peshawar, the provincial capital, has recently emerged as a focal point of dengue activity [1]. A detailed description of KP’s demographic, climatic, and dengue transmission profile, including its division into endemic and non-endemic zones, is available in previous studies [1,5].

### Study design

This is a cross-sectional study to investigate the entomological, virological, and epidemiological characteristics of dengue. Entomological surveys were conducted (in water-holding containers located indoors, outdoors, and on rooftops) in both dengue-endemic and non-endemic districts (Fig 1) during the most intense transmission period to explore potential associations of entomological and entomo-virological risk indicators with the cases that occurred during a dengue outbreak in KP (July–December, 2021). In order to acquire traditional larval indices or indicators for every district, we adopted the surveillance strategies recommended by [21]. Sample size (houses per district) varied, calculated using prior dengue prevalence, precision, and margins of error estimates [1,20], with details per district in S1 Table, influenced by district-specific factors including case number, human population density, geographic size, and local cooperation. When a house couldn’t be sampled in our surveys, either because no one lived there or the owner refused to participate, the next closest house was sampled. For the same period of corresponding entomological surveillance in a particular area, weekly dengue case data was collected from the dengue control cell office in the Directorate of Health Services (DHS) situated in Peshawar to explore any possible association between vector larval indices and dengue incidence occurring at each sampling area across the province. Here, endemic locations are those where dengue outbreaks have been observed for two successive years and have high incidence rates. An area with erratic urban expansion, low mean income, and higher *Aedes* mosquito density is referred to as being at high risk of dengue transmission. A suspected dengue case was defined as any patient with fever and at least two WHO-defined symptoms (e.g., joint pain/muscle, rash, or severe warning signs (e.g., persistent vomiting, mucosal bleeding) [22].

### Entomological surveillance

A trained team (2 males, 1 female) conducted larval surveys in districts reporting daily dengue cases exceeding three, previously identified as high-risk zones based on dengue prevalence data from the DHS [1]. Surveillance was supervised by medical entomologists. Each district’s survey covered designated union councils (administrative units), with a structured approach guided by a map and a list of target locations. Each day, at least 20 households were inspected per union council throughout the dengue transmission season. Collection sites included intra-domestic (inside homes), peri-domestic (patios, rooftops), and outdoor (public spaces, e.g., ponds) areas, with diverse water-holding containers (e.g., tires, tube wells). *Aedes* breeding containers were defined as any water-holding receptacle harboring immature stages of *Ae. aegypti* or *Ae. albopictus*. Each container and household were classified as positive if at least one *Aedes* larva was detected [23]. Before container inspection, householders were asked whether any container had been treated with insecticides or larvicides. None of the inspected containers had been chemically treated or cleaned in the preceding 72 hours. Larval collection was conducted during 07:00–13:00, following WHO (2011) guidelines for dengue vector surveillance [21,23]. For larval sampling, small water-holding containers (<50L) were emptied into a white tray, and the larvae were collected using a plastic Pasteur pipette. Large containers (>50L), such as plastic or metal drums, were sampled using ladles and aspirators. In case of high infestation (>20 larvae), water was drained through a sieve for collection [10]. Larval density was categorized into three levels: 0–10, 11–100, and >100, as suggested by Cromwell et al. (2017) [24]. A limitation of this study is the absence of a pupal survey, which could have provided additional insights into adult emergence. However, due to time and resource constraints, we focused on larval and adult collections to assess population dynamics. Additionally, adult *Aedes* mosquito sampling was done particularly during dusk (6–8 p.m.) and dawn (8–11 a.m.), with a backpack aspirator and nets in houses, wedding halls, canteens/hotels, and educational institutes, which are public venues where dengue cases were reported previously [24,25]. This can be used as a proxy for evaluating adult mosquito production for future targeted actions to control breeding in productive public places. All the samples, including the adults and larvae, were shipped to the study’s facility and further analysis was carried out as discussed below.

### Rearing and identification of mosquitoes

Collected samples were transported (on a daily basis) in lidded plastic containers to the entomology laboratory at the Nuclear Institute for Food and Agriculture (NIFA), Peshawar, and the Zoology Department at Abdul Wali Khan University Mardan (AWKUM), KP. Larval samples from each sampling spot and container type were reared individually in separate trays and labeled with essential information, including collection date, container type, and location, etc. Pupae from each rearing tray were collected twice daily (9 am and 5 pm) and transferred to individual cages for adult emergence. Both the larval rearing and adult maintenance was carried out according to our published protocols [26]. Emerging adults were morphologically identified using the published taxonomic keys [27,28], counted, sexed, and data on the species and number collected from the various container types, habitats, and sampling sites were recorded. Larval identification was made at the fourth instar larval stage. *Ae. albopictus* larvae are characterized by comb scales with a single denticle, whereas *Ae. aegypti* larvae have comb scales with distinct central and lateral denticles (S1 Fig) [28]. This means *Ae. aegypti*’s comb scales have a more complex structure, with projections in the center and on the sides, differentiating them from those of *Ae. albopictus*. Larvae or adults other than *Ae. aegypti* and *Ae. albopictus* were excluded from the study. The proportion of larvae or adults of each *Aedes* species was calculated as the number of specimens collected/identified divided by the total number of larvae/adults of that species in an area. Collected adult samples were preserved for future *Aedes* mosquito population genetics studies. *Stegomyia* or entomological indices were calculated following WHO (2009) [29] guidelines.

### Molecular investigations

#### Identification of DENV in mosquitoes.

To assess DENV serotypes, 200 field-collected adult mosquito pools (20 per district), with 100 each of *Ae. aegypti* and *Ae. albopictus*, were processed following [30,31]. Each pool contained 10 adult indoor mosquitoes from each district [25,32]. These mosquitoes were from dengue non-endemic and dengue-high-risk locations that meet the required sample size. Pools with initial negative PCR results (using universal primers D1/forward and D2/reverse) were not subjected to further DENV genotyping (DENV1–4) as per our previous protocols [32]. The DENV infection rate in mosquitoes was estimated using minimum infection rates (MIR), calculated as the number of positive pools by species/total number of that species tested, multiplied by 1000 [33].

#### Ribonucleic acid (RNA) extraction and Polymerase Chain Reaction (PCR).

RNA extraction and PCR procedures were performed following established protocols [32,34,35]. Briefly, RNA was extracted from mosquito samples using the Vazyme Total RNA extraction kit V2 (Cat: RC112–01), and serotyping was conducted with dengue-specific primers, along with positive and negative controls. Full details on primer, thermal cycling conditions, and validation procedures are provided in [32].

#### Statistical analysis and data interpretation.

Frequencies and percentages were used to present mosquito breeding habitats and *stegomyia* indices. House index (HI) was calculated by dividing the number of houses positive for *Aedes* larvae by the total number of houses surveyed multiplied by 100; Container index (CI) was calculated by dividing the number of containers positive for *Aedes* larvae by the total no. of containers multiplied by 100); and Breteau index (BI) was calculated by dividing the number of containers positive for *Aedes* larvae by the total number of inspected houses multiplied by 100. A choropleth map was used to present the district-wise distribution of *Aedes* mosquitoes positivity.

Dengue incidence was calculated by dividing the total number of dengue cases by the total population at risk, and then multiplying the result by 100,000. To examine the relationship between dengue incidence and demographic and climatic factors, we conducted an ecological negative binomial regression model due to the count-based nature of dengue case data and the presence of overdispersion (variance exceeding the mean). Data were aggregated for 22 districts, with both response and predictor variables representing district-level summaries. This model was chosen as it allows for greater variability than the Poisson model, making it more suitable for our data. The response variable for the regression model was dengue incidence (cases aggregated at each district), while the explanatory variables included population density, Stegomyia indices (HI, CI, BI), and climatic factors such as average air temperature (°C), total precipitation (mm/day), and relative humidity (%) at 2 meters above the surface. Data for these predictor variables were also aggregated at the district level. Population density data were sourced from the Pakistan Bureau of Statistics, and climatic data were obtained from the NASA Langley Research Center (LaRC) POWER Project (https://power.larc.nasa.gov). Mean values for these climatic variables were calculated and aggregated at the district level for analysis. Incidence Rate Ratios (IRRs) were calculated to quantify the strength of associations between explanatory variables and dengue incidence, providing a more intuitive understanding of the results. To reduce the risk of overfitting due to limited number of observations, only univariable regression was conducted and results are interpreted as exploratory. In addition to the regression analysis, Chi-square tests were performed to assess associations between larval indices and categorical factors such as container type and location, with the results reported using p-values and confidence intervals. All analyses, including both descriptive and inferential statistics, were conducted using R software. In the regression model, the Wald Test Statistic was used to assess the significance of individual predictors. Statistical significance was determined using a p-value threshold of 0.05.

## Results

### Larval habitats

**Fig 2:**
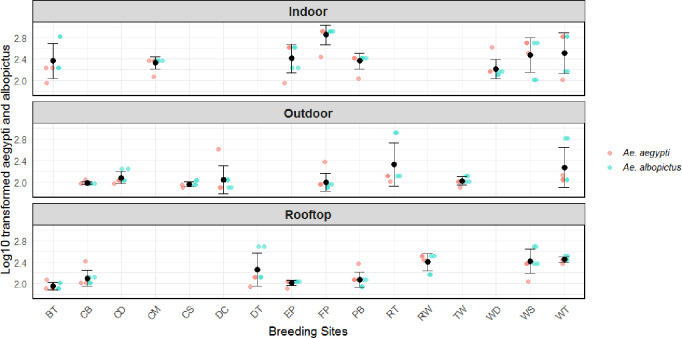
Summary of larval collection from multiple breeding sites/containers across KP, where the two letter subscripts represents: BT: Bucket, CB: Can & bottle, CD: Car wash dicks, CM: Cement basin, CS: Canals, DC: Disposable container, DT: Discarded Rubber tyres, EP: Earthen pot, FP: Flower pot, PB: Plastic bowls, RT: Rubber tyres, RW: Rooftop water, TW: Tube wells, WD: Water dispenser, WS: Water storing drums, WT: Water tanks. The y-axis represents the log10-transformed counts of larvae per container type. The black points indicate data points, while the colored points show mean values with 95% confidence intervals based on bootstrapped standard errors.

A total of 3,429 (20.4%), 443 (14.8%), and 631 (15%) containers were found positive for mosquito larvae from a total of 16,803 (indoor), 3,000 (outdoor), and 4,200 (rooftop) containers inspected, respectively (Fig 2). Flowerpots (25.4%) were the most important indoor breeding site, followed by water tanks (19.5%) and water storage metallic drums (14.7%). Among outdoor containers, rubber tires (16%) produced the highest number of larvae, followed by vehicle wash sites (shops) (14.8%) and tube wells (13.4%). On the rooftop, the stagnant water from dripping roof taps was the most alluring place for mosquitoes to reproduce (23.7%), followed by the water tank (18.6%) and the water storing metallic drum (14.8%).

### Larval composition in different breeding locations

**Fig 3:**
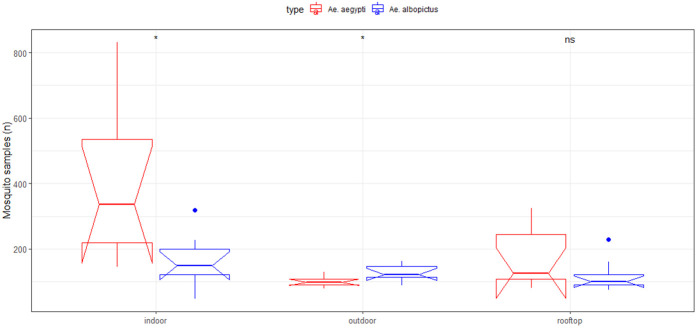
The box plot representing cumulative distribution of *Ae. aegypti* and *Ae. albopictus* larvae across different container placement (outdoors, indoors, and roof tops), irrespective of container type. The *P* values (at 5% level of significance) were computed using the Chi Square test statistic for independence. Notched Boxplots showing the significant differences in larval indices between *A. aegypti* and *A. albopictus*. The p-values represent the results of statistical tests comparing the two species, indicating inter-species differences rather than associations with external factors such as climatic conditions or container types. The boxes represent the interquartile range (IQR), the horizontal line inside each box represents the median, and the whiskers extend to the minimum and maximum values within 1.5 times the IQR. Outliers are shown as individual points. The notches represent approximate 95% confidence intervals for the medians, and the appearance of the lower quantile line being higher than the edges of the box is due to the notch design, not an error. The boxes represent the interquartile range (IQR), the horizontal line inside each box indicates the median, and the whiskers extend to the minimum and maximum values within 1.5 times the IQR.

Overall, a total of 8,683 larvae were recovered, yielding *Ae. aegypti* (5591; 62%) and *Ae. albopictus* (38%) (Fig 2). Of the larvae collected, 52% were from indoor water containers, followed by 21% from outdoor and 27% from rooftop sources. Indoor inspections collected a total of 3,209 (71%) *Ae. aegypti* and 1,311 (29%) *Ae. albopictus* larvae. Outdoor collections yielded 803 (44%) *Ae. aegypti* and 1,022 (56%) *Ae. albopictus* larvae. Rooftops surveillance found 1,379 (59%) *Ae. aegypti* and 959 (41%) *Ae. albopictus* larvae. Moreover, among the indoor and roof-top surveillance, we observed a significant association between the mosquito’s (both *Ae. aegypti* and *Ae. albopictus*) breeding choice and the type and location of container (P < 0.00) (Fig 3). However, the association was not significant for outdoor container types and larval breeding choice, which means larval breeding was independent of the type of container outside (P < 0.46).

### *Stegomyia* indices

**Fig 4:**
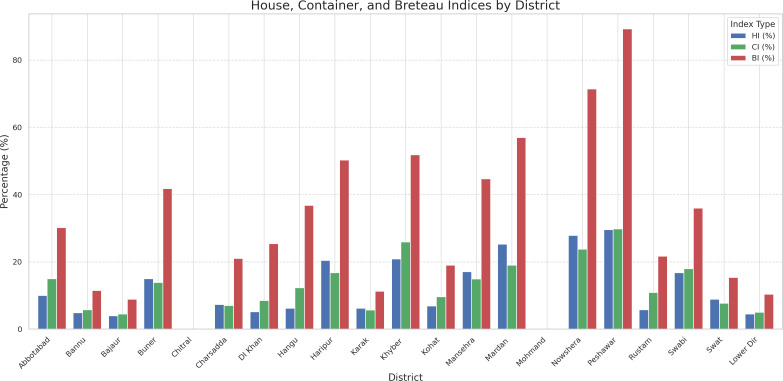
*Stegomyia* indices across various districts in the province.

Among the total 6,342 houses surveyed, only 1,232 were found positive for *Aedes* larvae, with a HI of 19.4% (Fig 4 and S1 Table). Among the total inspected containers (n = 16,803), only 3,429 were positive, with a CI of 20.4%. Maximum BI values were documented for Peshawar (n = 89), Nowshera (n = 71), and Mardan (n = 57). Peshawar, with an HI of 29.6%, was found to be highly infested, followed by Nowshera (27.8%) and Mardan (25.3%). Monthly analysis showed higher larval abundance in October (29.3%) and September (24.7%), with corresponding increases in adult mosquito populations of 24.7% and 20.2%, respectively. Collections of adult mosquitoes yielded a total of 10,393 adults, comprising *Ae. aegypti* (67.8%) and *Ae. albopictus* (34.2%) with a significant difference (p < 0.000) (Table 1). Female mosquitoes represented about 63% of the entire sample, with no discernible differences based on sampling between site/district. Universities and warehouses, with favorable mosquito breeding habitats, produced a significant proportion of adult mosquitoes (21% and 17.7%, respectively).

**Table 1:** Entomological indicators and dengue incidence during the July–December 2021 outbreak in KP.

Entomological investigations							Dengue incidence				
**Month**	**Inspected** **Containers (n)**	**Positive** **Containers** **(n)**		**Larval Infestation (%)**		**Adult** **Collection (%)**	**Suspected** **Cases** **(n)**	**Confirmed** **Cases** **(n)**		**Incidence (%)**	**Death** **(n)**
July	3578	361		10		1226 (11.8)	949	85		8.95	0
Aug	3792	512		13.5		1487 (14.3)	1750	262		14.9	0
Sept	3993	934		23.4		2567 (24.7)	2908	1363		46.8	0
Oct	4720	1381		29.3		2099 (20.2)	6639	3512		52.8	6
Nov	5213	1176		22.6		1788 (17.2)	148	65		43.9	4
Dec	2707	139		5.2		1226 (11.8)	51	3		5.8	0
**Total**	**24003**	**4503**		**18.8**		**10393**	**12445**	**5290**		**42.5**	**10**
χ^**2**^**-718.52, DF-5, P < 0.000**						χ^**2**^**-636.59, DF-5, P < 0.000**					
**Abundance of adult *Aedes* mosquito species in multiple places**											
**Habitat**			***Ae. aegypti* (n) (%)**		***Ae. albopictus* (n) (%)**		**Total (n)**		**Percentage**		
Houses			1529 (82.6)		321 (17.4)		1850		17.8		
Wedding halls			478 (57.4)		355 (42.5)		833		8		
Universities			1330 (60.4)		871 (39.6)		2201		21		
Colleges			744 (65.8)		387 (34.2)		1131		10.9		
Cattle market			691 (54.3)		581 (45.7)		1272		12		
Warehouse			1223 (66.6)		613 (33.4)		1836		17.7		
Canteens/Hotels			851 (67)		419 (33)		1270		12		
**Total**			**6846 (67.8%)**		**3547 (34.2%)**		**10393**				
**χ**^**2**^ **-363.979, DF-6, P value-0.000**											

P-values were derived from chi-square tests for categorical variables and t-tests for continuous variables to assess the significance of differences between groups. Values in brackets represent percentages of the total for the respective category. Where no brackets are used, the values represent whole numbers. A p-value < 0.05 was considered statistically significant.

### *Aedes* species composition across the province

**Fig 5:**
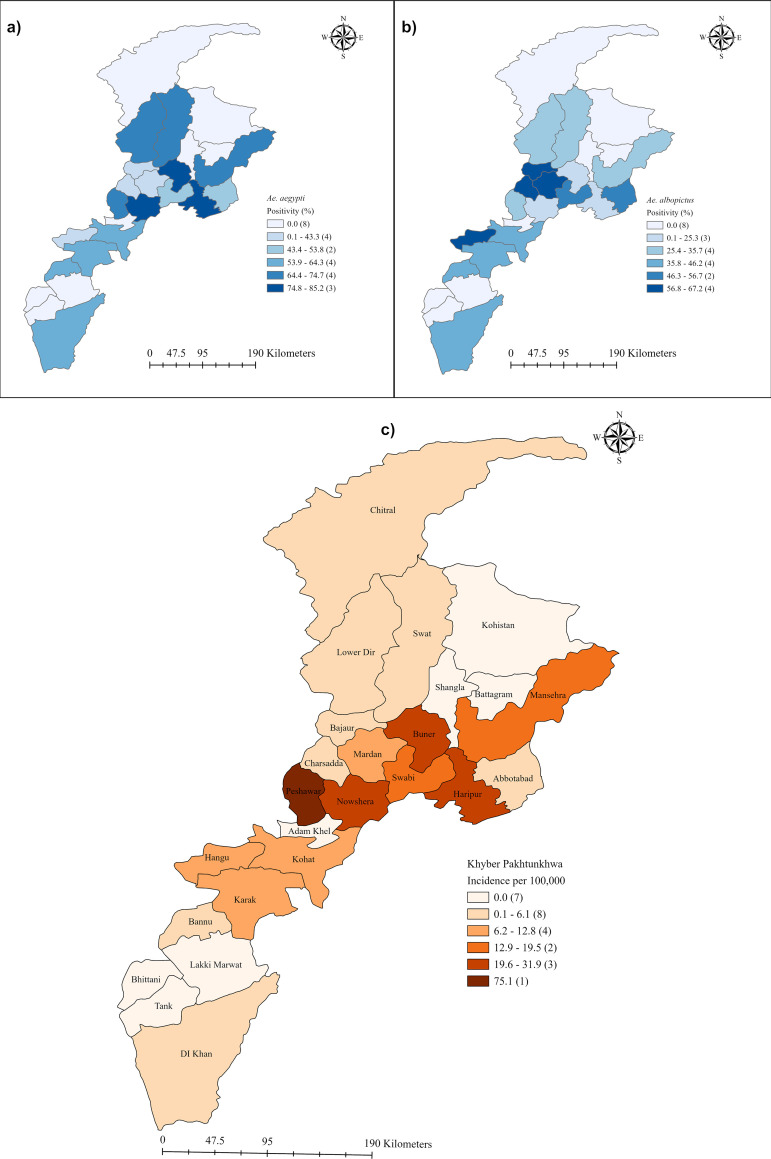
Maps showing the distribution of *Ae. aegypti* (panel a, left) and *Ae. albopictus* (panel b, right) (adults and larvae) across Khyber Pakhtunkhwa Province. Panel (c) illustrates dengue incidence per 100,000 population in the province. The shapefile for these maps was sourced from DIVA-GIS (https://diva-gis.org/).

The data indicate that both *Ae. aegypti* and *Aedes albopictus* were present in all parts of the province, though their proportion varied across different districts (Fig 5). The abundance of *Ae. aegypti* was notably higher in highly urbanized and developed districts, such as Peshawar, Kohat, and Nowshera, etc., which feature dense human populations, minimal vegetation, and slightly warmer climates. In contrast, *Ae. albopictus* was more prevalent in regions like Abbotabad, Bajaur, Charsadda, Rustam, and Swabi, characterized by denser vegetation and extensive irrigation lands. These regions, with their denser foliage and extensive water bodies, offer a suitable habitat for *Ae. albopictus*, which prefers more rural or semi-urban environments with abundant natural water sources. However, no *Aedes* mosquitoes were collected (during this study period) in the districts of Chitral and Mohmand, even though these districts documented some imported dengue cases (n = 15).

### Association between dengue incidence, population density, and entomological variables

Our analysis revealed an overall incidence of dengue of 20 per 100,000 population. The district-wise investigations revealed that areas with higher *stegomyial* indices, such as Peshawar (75.1 per 100,000), Haripur (31.9 per 100,000), Buner (29.2 per 100,000), and Nowshera (26.6 per 100,000), had higher dengue incidences (Fig 5). Moreover, our regression analysis demonstrated that dengue incidence was associated with population density, stegomiyal indices, and the high prevalence of *Ae. aegypti* (Table 2)*.* For each additional person per square kilometre, the incidence rate of dengue increased by 0.1%, holding the total population constant. The incidence rate of dengue increased by 11.7%, 11.2%, and 4.0% for each unit increase in the CI, HI, and BI, respectively. Likewise, an additional increase in the prevalence of *Ae. aegypti* was associated with an increase in the incidence rate of 2.8%.

**Table 2:** Regression analysis of the association between dengue incidence, population density, and stegomyia indices.

Parameters	Incidence rate ratio	95% CI	p value
Population density	1.001	1.001 - 1.002	0.035
CI	1.117	1.075 - 1.160	0.000
HI	1.112	1.076 - 1.150	0.000
BI	1.040	1.027 - 1.052	0.000
*Ae. aegypti* abundance	1.028	1.010 - 1.047	0.002
*Ae. albopictus* abundance	0.977	0.944 - 1.011	0.188

### Dengue virus detection in *Aedes* mosquitoes

Only 38 (19%) out of 200 mosquito pools (*Ae. aegypti* and *Ae. albopictus*, 18 and 20 pools, respectively) were found positive for DENV serotype-2 (47.4%), 3 (47.4%), and 1 (5.2%) (Table 3). Overall, the DENV infection rate among mosquitoes was 19% (MIR = 19). The peak MIRs were obtained for the districts of Peshawar (MIRs = 30), Nowshera, and Haripur (MIR = 25), whereas the lowest MIR was obtained for Kohat (MIR = 5).

**Table 3:** Identification and infection rates of dengue virus in adult *Aedes* mosquito species.

District	MP*	N	DENV-I		DENV-II		DENV-III		DENV-IV		Total	%	MIR*
			*Ae.aegypti*	*Ae.albopictus*	*Ae.aegypti*	*Ae.albopictus*	*Ae.aegypti*	*Ae.albopictus*	*Ae.aegypti*	*Ae.albopictus*			
Peshawar	20	200	1	–	1	1	2	1	–	–	6	30	30
Nowshera	20	200	–	–	1	1	2	1	–	–	5	25	25
Mardan	20	200	–	–	1	2	–	1	–	–	4	20	20
Haripur	20	200	–	1	–	1	1	2	–	–	5	25	25
Mansehra	20	200	–	–	1	1	2	–	–	–	4	20	20
Swabi	20	200	–	–	1	1	–	2	–	–	4	20	20
Buner	20	200	–	–	1	1	–	1	–	–	3	15	15
Khyber	20	200	–	–	1	1	2	–	–	–	4	20	20
Charsadda	20	200	–	–	–	1	–	1	–	–	2	10	10
Kohat	20	200	–	–	1	–	–	–	–	–	1	5	5
**Total**	**200**	**2000**	**1**	**1**	**8**	**10**	**9**	**9**	**0**	**0**	**38**	**19**	**19**
**Total positive Pools**			**2**		**18**		**18**		**0**				

*MP, Mosquito Pool; *MIR, Minimum Infection Rates.

## Discussion

Our study in KP Province provides critical insights into the entomological and virological factors driving dengue transmission. *Ae. aegypti* dominated both larval (62%) and adult (67.8%) collections, with significant presence in urbanized districts like Peshawar, Nowshera, and Mardan, where Breteau Indices (BI) reached 89, 71, and 57, respectively. *Ae. albopictus*, comprising 38% of larvae, was more prevalent in semi-rural areas with denser vegetation, such as Abbottabad and Charsadda. The co-occurrence of these species, alongside high larval indices (HI = 19.4%, CI = 20.4%, BI = 89%) and a 19% dengue virus (DENV) infection rate in mosquito pools (MIR = 19), underscores a substantial risk of dengue outbreaks in the region.

### Larval habitats and vector distribution

Among the indoor water containers, the highest larval infestation was observed in the water tanks, followed by flowerpots and water-storing metallic drums (Fig 2). Rubber tires were the most common outdoor container, followed by car wash sites and tube wells. Our findings align with previous national and international studies [8,25,32,36,37,38]. Water drums and tanks are large open-mouthed containers that hold important amounts of continuously available water that are never emptied and replaced on a regular basis. Uncovered containers, both outside and on rooftops, often collect rainwater, creating permanent *Aedes* mosquito breeding sites [39–41]. Rooftop tap water (23.7%) emerged as a significant site, likely due to persistent leakage creating stable breeding conditions. The significant association between container type/location and larval abundance indoors and on rooftops (P < 0.00) highlights the influence of human-modified environments on vector proliferation. Conversely, the lack of association outdoors (P < 0.46) suggests *Ae. albopictus* adapts to diverse peri-domestic conditions, consistent with its ecological flexibility [37,42]. These findings align with regional studies [25,32] identifying domestic containers as key *Aedes* habitats, emphasizing the need for targeted source reduction. However, our study did not assess container productivity (e.g., pupal output), limiting insights into adult emergence rates—a critical factor for transmission dynamics. Notably, while *Ae. aegypti* historically dominated [2,25], *Ae. albopictus* now predominantly proliferates in districts like Charsadda, Swabi, and Abbottabad (Fig 5), signalling a dual-vector challenge for control strategies. The role of socio-economic factors such as waste management and household water storage habits in breeding site availability should be explored further to improve vector control efforts.

### Entomological indices and dengue incidence

Our findings reveal a strong correlation between Stegomyia indices and dengue incidence, with Peshawar (75.1 per 100,000) and Nowshera (26.6 per 100,000) showing high case rates alongside elevated BI values (89 and 71, respectively). Regression analysis confirmed significant associations, with incidence rate ratios increasing by 11.7%, 11.2%, and 4.0% per unit rise in CI, HI, and BI, respectively (Table 2). Population density further amplified incidence (0.1% per person/km^2^), reflecting urbanization’s role in vector-human contact. However, the predictive reliability of these indices remains uncertain due to potential confounding factors. Bowman *et al*. (2014) reviewed studies with mixed findings; some [43–46] found significant positive relationships, supporting vector indices as predictive tools, while others [47,48] reported no significant associations, likely due to vector dispersal, human movement, sociodemographic factors, and secondary vector presence. These inconsistencies underscore the limitations of larval indices in outbreak prediction. Notably, our HI (19.4%) and BI (89) exceed the reviewed thresholds (e.g., BI = 5) [8,43], contrasting with weak correlations found elsewhere [47,49,50]. Our larval-only data, lacking pupal indices, limits causal inference, a gap noted as critical [8], favouring pupal/adult metrics. Future studies should integrate pupal surveys, adult mosquito surveillance, climatic factors, and human mobility across outbreak phases to establish predictive thresholds, adapting approaches from studies [7,51,52].

### Seasonal dynamics and transmission risks

Larval positivity peaked in September (24.7%) and October (29.3%), aligning with 46.8% and 52.8% of dengue hospitalizations, reflecting a post-monsoon surge [2,20,25,36]. *Ae. aegypti* likely drives urban peaks, while *Ae. albopictus* thrives in semi-rural areas [20,25,53]. This mirrors Pakistan’s seasonal trends [3,5,20], with climatic influence noted globally [54]. Limited to the transmission season, our study lacks dry-season data, precluding full seasonal analysis. Nonetheless, the temporal overlap between vector abundance and case peaks supports climatic influence on transmission, warranting further investigation into environmental drivers (e.g., humidity, temperature) and their interaction with vector ecology. Future studies should include year-round surveillance to model climatic impacts and enhance outbreak prediction in the region.

### Molecular insights into DENV circulation

The rate of virus infection in the vector may serve as a reliable epidemiological indicator of dengue risk within a given locality, informing targeted vector control strategies [8]. Of 200 mosquito pools, 38 (19%) tested positive for DENV, with DENV-2 and DENV-3 predominant (47.4% each; Table 3), indicating active epidemic transmission by *Aedes aegypti* and *Ae. albopictus* in KP during 2021. The higher MIR in Peshawar (30) compared to Kohat (5) aligns with dengue incidence rates (75.1 vs. ~ 5/100,000) (Fig 5), suggesting a spatial risk gradient. Our MIRs, higher than those reported in Lahore (0.75–5.1) [55] but lower than peak values in Swat [25,36], suggest that dengue transmission does not occur at a fixed entomological threshold but rather fluctuates based on multiple factors, including seroprevalence, mosquito density, and climatic conditions [45]. Co-circulation of these serotypes heightens severe disease risk via antibody-dependent enhancement [56], a public health concern. However, lacking genomic or transovarial data, this cross-sectional study limits our ability to determine viral origins or persistence. Longitudinal genomic surveillance is essential for predicting future outbreaks and informing targeted vector control strategies.

### Critical contextualization and limitations

Our findings extend prior similar studies [2,57] by mapping larval habitats and DENV infection across multiple districts, highlighting the expansion of *Ae. albopictus* alongside *Ae. aegypti*. However, limitations temper our conclusions. The absence of pupal surveys restricts adult production estimates, while underreported cases and human mobility data gaps may bias incidence associations. Sampling constraints (e.g., single-season focus) and untested environmental variables further limit generalizability. Additionally, due to limited data, we were unable to perform a fully adjusted multivariable regression analysis, which may affect the interpretation of individual associations. While univariable analyses provided useful insights, we acknowledge that these variables are not independent and should be interpreted with caution. Future studies with larger datasets should consider multivariable approaches to account for potential confounders and interdependencies among variables.

### Public health implications

This study provides a robust baseline for dengue risk in KP, identifying high-risk districts and key breeding containers for targeted control. The urban dominance of *Ae. aegypti* and the adaptability of *Ae. albopictus* present a dual-vector challenge, heightening arboviral transmission risks. Public health strategies should prioritize eliminating flowerpots, tires, and rooftop water sources, particularly in Peshawar, where indices and MIRs peak. Strengthening community engagement and routine entomological and virological surveillance will enhance cost-effective interventions. By linking larval indices, DENV infection, and incidence, our data inform evidence-based policies to mitigate outbreaks in this endemic region.

## Conclusions

Our findings revealed significant differences in mosquito breeding sites for *Aedes* species and identified potential dengue transmission areas. This information can establish a baseline for predicting dengue outbreak risks, especially in densely populated cities where DENV-2 and DENV-3 circulate. Our results provide actionable insights for policymakers to develop targeted guidelines aimed at curbing the rising trends of dengue in KP. Public health initiatives should prioritize high-risk areas identified through vector indices and incorporate community engagement in eliminating potential breeding sites. To ascertain the reported differential risk patterns, additional investigation is essential. Further investigations into the relationship between vector index levels, MIRs, and dengue incidence in various geographic locations across the province/country will be essential and helpful in developing vector management strategies such as *Wolbachia* technology. Such information and evidence empower communities to identify and manage breeding sites, enhancing dengue prevention efforts by targeting key containers that produce most adult *Aedes* mosquitoes and enabling cost-effective, site-specific control programs.

## Supporting information

S1 Fig Morphological Identification of *Ae. aegypti* and *Ae. albopictus* Larvae. (DOCX)

S1 Table Household Indoor Larval Surveillance and Reported Dengue Cases in Khyber Pakhtunkhwa Province, July–December 2021. (DOCX)
