# Supplementary material for: Assessing Aedes mosquito larval indicators, dengue virus infection rates, and risk factors in Khyber Pakhtunkhwa: Insights for improved vector control strategies
Source: PLoS Negl Trop Dis. 2025 Jul 22;19(7):e0013252. doi: 10.1371/journal.pntd.0013252 (PMC12306792; doi:10.1371/journal.pntd.0013252)
Supplement: S1 Fig — (DOCX) [file pntd.0013252.s001.docx]

**
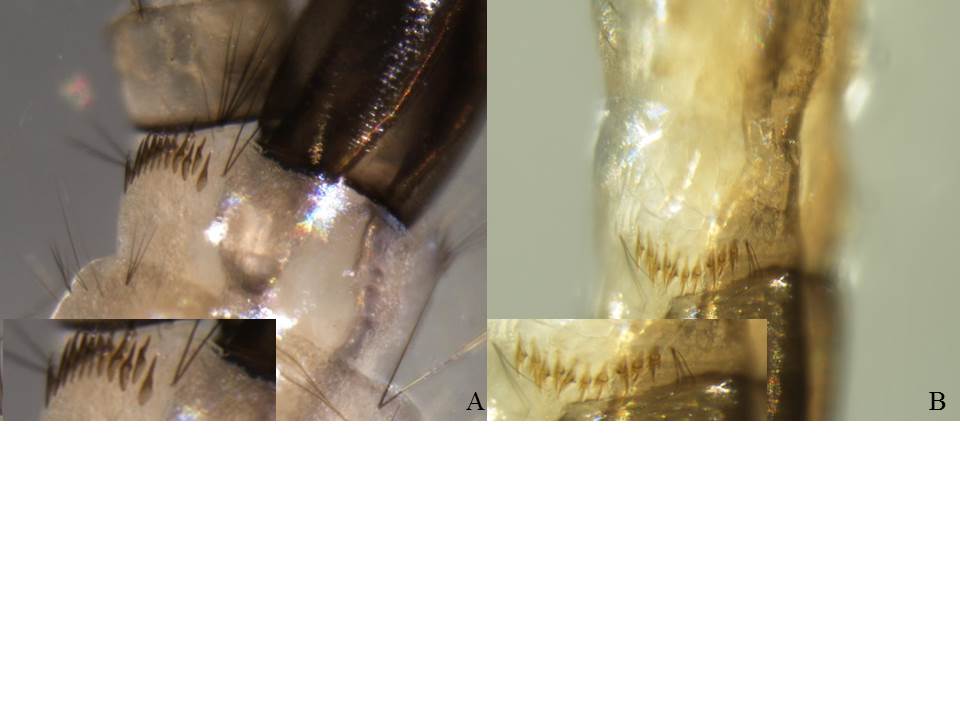
**

**Figure 1**: **Identification of *Aedes* larvae.** A) Ae. albopictus larvae with comb scales and a single denticle. B) *Ae. aegypti* larvae with distinct central and lateral denticles on the comb scales.
