# Supplementary material for: Assessing Aedes mosquito larval indicators, dengue virus infection rates, and risk factors in Khyber Pakhtunkhwa: Insights for improved vector control strategies
Source: PLoS Negl Trop Dis. 2025 Jul 22;19(7):e0013252. doi: 10.1371/journal.pntd.0013252 (PMC12306792; doi:10.1371/journal.pntd.0013252)
Supplement: S1 Table — (DOCX) [file pntd.0013252.s002.docx]

**Table 1: Household indoor larval surveillance and dengue case load during July–December, 2021**

**Test statistics**: Inspected houses (χ^2^-379.156, DF-19, P<0.000), and Inspected containers (χ^2^-828.318, DF-19, P<0.000)

**House index (HI):** (no. of houses positive for aedes larvae/total no. of houses surveyed x 100)

**Container index (CI):** (no. of containers positive for aedes larvae/total no. of containers x 100)

**Breteau index (BI):** (no. of containers positive for aedes larvae/total no. of inspected houses x 100)
